# Clinical considerations at the intersection of hematopoietic cell transplantation and hereditary hematopoietic malignancy

**DOI:** 10.3389/fonc.2023.1180439

**Published:** 2023-05-12

**Authors:** Timothy E. O’Connor, Reid Shaw, Rafael Madero-Marroquin, Gregory W. Roloff

**Affiliations:** ^1^ Department of Medicine, Loyola University Medical Center, Maywood, IL, United States; ^2^ Section of Hematology/Oncology, The University of Chicago, Chicago, IL, United States

**Keywords:** hereditary hematopoietic malignancy, AML, MDS, genetic testing, stem cell transplant

## Abstract

In recent years, advances in genetics and the integration of clinical-grade next-generation sequencing (NGS) assays into patient care have facilitated broader recognition of hereditary hematopoietic malignancy (HHM) among clinicians, in addition to the identification and characterization of novel HHM syndromes. Studies on genetic risk distribution within affected families and unique considerations of HHM biology represent exciting areas of translational research. More recently, data are now emerging pertaining to unique aspects of clinical management of malignancies arising in the context of pathogenic germline mutations, with particular emphasis on chemotherapy responsiveness. In this article, we explore considerations surrounding allogeneic transplantation in the context of HHMs. We review pre- and post-transplant patient implications, including genetic testing donor selection and donor-derived malignancies. Additionally, we consider the limited data that exist regarding the use of transplantation in HHMs and safeguards that might be pursued to mitigate transplant-related toxicities.

## Introduction

It is now well understood that inherited pathogenic mutations contribute to about 10% of newly diagnosed solid tumors (1). The prototypical familial cancer syndrome described by Li and Fraumeni in the 1960s involving neoplasia of the breast, bone, brain, and adrenal glands jumpstarted the nascent field of cancer genetics (2). The first case of hereditary hematopoietic malignancy was detailed in 1922 and ultimately published in series by Gunz and colleagues in 1975. Their description spanned 909 families, identifying 72 individuals with leukemia who had one or more relatives who were also affected with leukemia (3). In 1999, the autosomal dominant familial platelet disorder with propensity to develop acute myeloid leukemia (now known as *RUNX1* Familial Platelet Disorder with Associated Myeloid Malignancies, *RUNX1*-FPDMM) was the first HHM to have its molecular basis elucidated, driven by heterozygous pathogenic *RUNX1* variants (4). More than a dozen genes have been subsequently identified as direct drivers of HHMs, with multiple others identified as conferring risk of blood cancer as one phenotypic element within broader solid tumor predisposition syndromes (5, 6).

Although understandably anxiety provoking, the identification of a familial cancer syndrome can also prove empowering to patients. First, it provides knowledge to guide the evaluation of seemingly unaffected family members of the index patient via cascade testing. Because some variants confer risk to more than one tumor type, verifying the presence of a pathogenic germline variant streamlines opportunities for screening and surveillance of other malignancies and disease phenotypes (6). Precision medicine treatment approaches have also emerged and entered the standard of care for common tumor types, such as the use of PARP-inhibitor therapy in germline BRCA-mutant breast cancer (7). Unlike solid tumors, in HHMs the mutation status of family members may influence decision making for the care of the index patient due to considerations of donor source when hematopoietic cell transplant (HCT) is pursued. Additionally, distinct biology of HHM syndromes also influences transplant conditioning regimens and strategies for toxicity mitigation in a vulnerable patient population. In this article, we review high-yield HHM syndromes that are relevant to HCT and consider the place for HCT in disease management strategies. An overview of HHM testing is provided and data regarding HCT outcomes in established HHM syndromes are presented.

## Overview of select HHMs

Currently, pathogenic germline variants in *DDX41* mutations represent the most common cause of HHMs, representing approximately 2.4% of patients in a large cohort study of 1385 individuals with AML or MDS (8, 9). Development of myeloid malignancy in patients with germline *DDX41* mutations occurs at an age that approximates the median age of onset for *de novo*/sporadic AML/MDS. Most affected individuals demonstrate normal blood counts well into adulthood, suggesting that *DDX41* haploinsufficiency is sufficient to support baseline hematopoiesis (9). However, loss of heterozygosity leads to the development of cytopenias and macrocytosis at a mean age of 66 years (range 50-85 years) and hematologic malignancy shortly thereafter with a mean age at diagnosis of 69 years (range 36-88 years. Myeloid malignancy associated with *DDX41* variants is felt to be highly treatment responsive. In 20 patients with MDS/AML treated with chemotherapy (n=9) or azacitidine (n=11), response rates were 100% and 73%, respectively, and a median survival of 5.2 years was observed (8). Another study among 3132 unrelated adult patients with myeloid malignancies identified 28 individuals (20/28 male) with germline *DDX41* variants diagnosed with AML described chronic clinical trajectories (mean duration 11.2 months) during which patients with cytopenias were monitored before AML onset. When diagnosed, 69% of patients had acquired a second hit (somatic) *DDX41* variant and most cases had a normal karyotype (75%) with bone marrow hypocellularity (93%) (10). Recently, in a large, multinational analysis of myeloid neoplasia in 9082 patients, 346 patients with pathogenic/likely pathogenic germline and/or somatic *DDX41* variants were identified. Statistically, it was calculated that about 80% of myeloid malignancy with germline risk contribution identified in the study population was attributable to *DDX41* mutations alone. Risk variants were enriched within Japanese patients by 10-fold when compared to those from other geographic areas. The lifetime risk of acquiring myeloid malignancy in the population by age 90 was 49%. Moreover, co-mutational status, both at diagnosis and at time of disease progression did not affect clinical outcomes, even in the case of multi-hit *TP53* variants, suggesting that *DDX41*-driven myeloid malignancies comprise a unique subset of myeloid malignancy with distinct molecular biology (11).

Biallelic *CEBPA* mutations are found in approximately 10% of patients with AML. It is estimated that as many as ten percent of these (~1% total cases) may represent familial variants (12). Familial *CEBPA*-mutated AML was first described in 2004, with three family members harboring identical N-terminal germline mutations (13). AML development generally occurs when secondarily acquired C-terminal mutations arise, typically at an average age of onset of 24.5 years (range 2-46 years) and with affected kindreds demonstrating >90% penetrance for AML diagnosis (13–15). Due to high chemotherapy responsiveness, familial *CEBPA*-mutated AML generally portends a favorable prognosis in comparison to cases with sporadic mutations, with 10-year survival rates of 67%, superior to spontaneous bi-allelic (54%) and single *CEBPA* mutations (29%) (15). However, as discussed more below, recurrent leukemic episodes are common and consideration of allogeneic replacement of a stem cell pool harboring pathogenic germline variants should be considered once remission is achieved.

Autosomal dominant mutations in *GATA2* lead to the well-described GATA2 deficiency syndrome. Although the clinical phenotype can vary, the syndrome harbors several notable phenotypic features that must be recognized by hematologists. First, flow cytometric profiling of affected patients often demonstrates substantial reduction or near absence or B-lymphocytes, NK cells, and monocytes, leading to immunodeficiency (16, 17). Recurrent infections manifest throughout late childhood and adolescence. Disseminated nontuberculous mycobacterial, invasive fungal, human papillomavirus infections should warrant dedicated genetics investigation. Additionally, limb lymphedema, deafness, and pulmonary proteinosis also contribute to disease phenotype. This severe immunodeficiency, which typically presents in adolescence, leads to a variety of disseminated infections, including nontuberculous mycobacterial, invasive fungal, and disseminated human papilloma virus infections (18). Finally, *GATA2* deficiency confers risk to the development of myeloid malignancy, typically in the form of MDS/AML (19). HHMs driven by germline *GATA2* mutations generally have poor overall survival when analyzed by germline variants alone, but this affect appears to be equalized when accounting for karytotypic/cytogenetic features (20).

**Figure 1:**
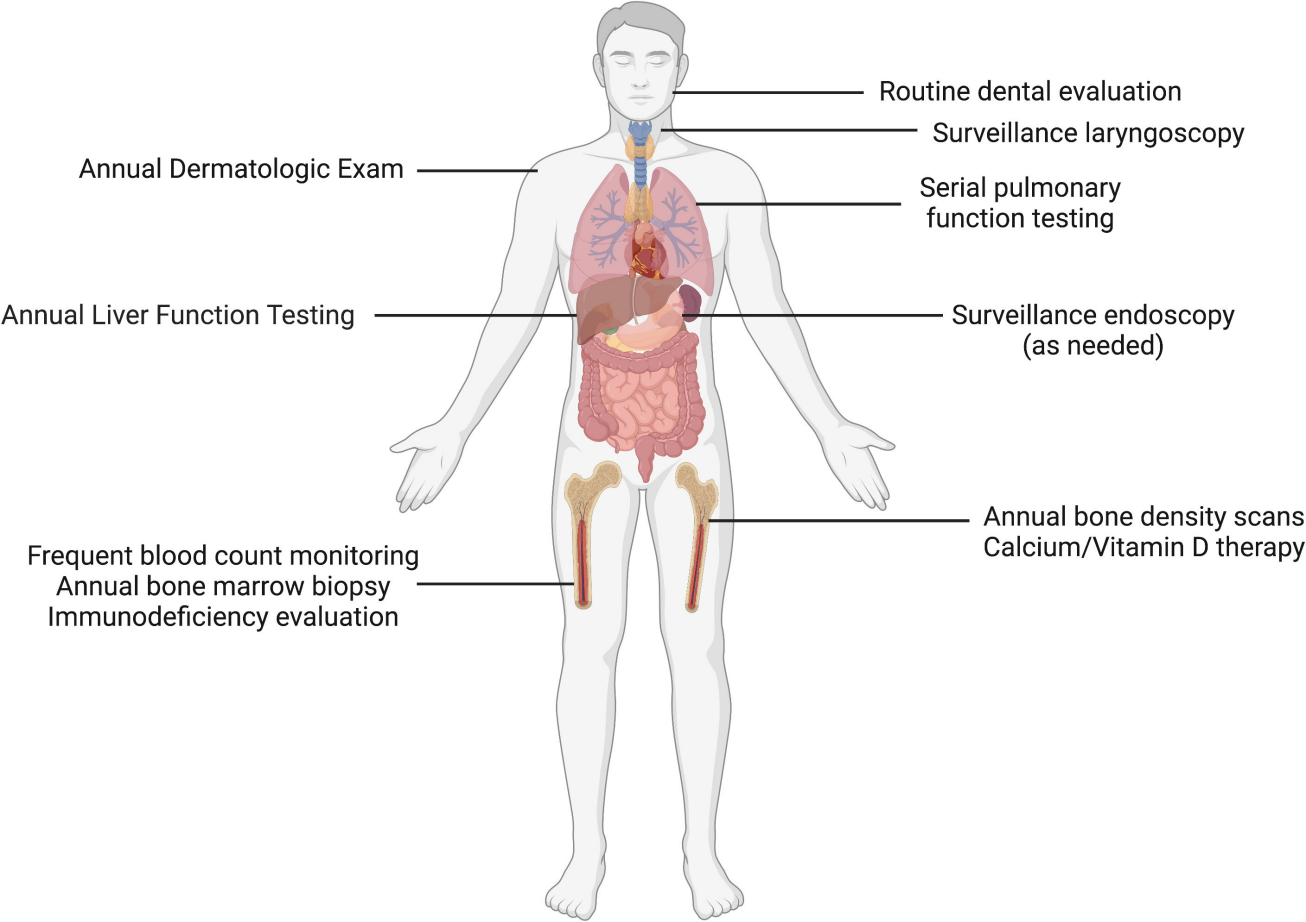
Clinical surveillance in short telomere syndromes. Patients with short telomere syndromes are at highest risk for bone marrow failure, pulmonary fibrosis, liver disease and solid cancers. Surveillance strategies implemented within a multidisciplinary cancer risk program are aimed to identify characteristic disease phenotypes and facilitate timely intervention. These abnormalities include such as cytopenias/bone marrow failure, interstitial lung disease, cryptogenic cirrhosis, and squamous cell carcinomas, especially of the tongue. Image made with BioRender.com.

Familial platelet disorder with predisposition to myeloid malignancy (FPDMM), sometimes referred to as Thrombocytopenia 2, was first described in 1985 and molecularly characterized in 1999 as the result of pathogenic variants in the transcription factor *RUNX1* (4). A wide spectrum of variants has been described, including multiexon or whole gene deletions, frameshifts, missense and nonsense mutations. Strikingly, clonal hematopoiesis has been observed in a majority of asymptomatic *RUNX1* germline variant carriers, even before age 50. The lifetime risk of developing leukemia is estimated to be ~35% (21, 22). *RUNX1*-mutated AML is associated with dismal relapse-free and overall survival when compared to controls with *RUNX1* wild type status (23). Short telomere syndromes (STS) are characterized by accelerated aging, and associated with mutations in genes that maintain the “molecular clock,” including TERT, TERC, and DKC1 (24–26). The loss of function of these genes lead to a variable phenotypic presentation that includes non-hematopoietic phenotypes including idiopathic pulmonary fibrosis (27), premature graying of hair, cryptogenic cirrhosis, and hematologic consequences including aplastic anemia, MDS, and AML (28, 29). Because the clinical phenotype of STS involve multisystem disorders, clinical surveillance is imperative and patients benefit from involvement of multispecialty teams with experience in cancer risk assessment (**Figure 1**) (30).

Unlike familial CEBPA-mutated AML, wherein AML development appears to be the principal clinical feature, other inherited cancer syndromes associated with HHMs and hematopoietic phenotypes also confer solid tumor risk. As mentioned above, the first hereditary cancer syndrome described by Li and Fraumeni detailed kindreds with sarcoma, leukemia, and cancers of the brain, breast, and adrenal gland (2). Hypodiploid ALL is the most common hematopoietic malignancy reported to be associated with Li-Fraumeni syndrome (LFS), although cases of AML and chronic myeloid malignancies such as myelodysplastic syndromes and chronic myeloid leukemia have also been described. Unfortunately, large datasets with carefully annotated clinical trajectories for hematopoietic malignancy in Li-Fraumeni syndrome (LFS) patients remain scarce. In a series of 7 LFS patients who received care for hematopoietic malignancy at MD Anderson Cancer Center (31), five patients underwent HCT. Only one of these five patients, an individual with B-cell ALL, remained in durable remission at least one year after transplantation. Five of the seven patients had malignancies that were classified as therapy-related myeloid neoplasms and experienced dismal outcomes (31). This LFS experience underscores the importance of hematopoietic malignancy risk assessment in patients who have received prior cancer therapy, with particular emphasis on patients with inherited cancer predisposition syndromes. As Churpek and colleagues have identified previously undiagnosed germline mutations in 10/47 (21%) of breast cancer patients who developed therapy-related leukemia, it is important to recognize that the discovery of an inherited predisposition syndrome may not occur until subsequent malignancies develop after an initial cancer diagnosis (32).

Further evidence towards characterizing solid tumor manifestations of HHM-associated germline syndromes is emerging. For example, in seminal studies that established germline variants in *DDX41* as HHM-associated genes, 6/33 (18%) individuals with blood cancers had received prior solid tumor diagnoses, with prostate cancer being the most common (3/33, 9%) (8, 9, 33). However, isolated solid tumors in germline variant carriers were also reported in the absence of hematopoietic malignancy, and it is not known whether solid tumor incidence is higher in patients with germline DDX41 variants than would be expected in the general population (5). **Table 1** describes cardinal hematopoietic and non-hematopoietic clinical features of the above-discussed HHMs, and summarizes published HCT experiences.

**Table 1:** High-yield characteristics of hereditary hematopoietic malignancies and stem cell transplant considerations.

Gene	Hematopoietic Phenotype	Non-Hematopoietic Phenotype	Transplant Considerations	Citations
*CEBPA*	AML	none	Transplant-associated morbidity vs. toxicity of repeat chemotherapy exposure for subsequent leukemic episdes	(15, 34)
*CHEK2*	CLL, MDS, AML	Breast, prostate, thyroid, colorectal, pancreatic, renal and thyroid carcinoma	No robust data; Consideration of donor testing if using related donor source	(35)
*DDX41*	MDS, AML,	Solid tumor risk?	Severe acute GVHD post-transplant; Post-transplant Cy preferred for GVHD prevention	(36, 37, 38)
*ETV6*	B-ALL, MDS, AML	none	No robust data; Avoidance of matched related donors with unknown carrier state	(39, 40)
Fanconi genes	MDS, AML, ALL	Carcinoma of the oral/squamous epithelium and GI-tract, short stature, facial dysmorphology, skeletal/digit anomalies, hypogonadism	Reduced-intensity conditioning preferred due to elevated toxicity of radiation and alkylating agents	(41, 42, 43, 44)
*GATA2*	MDS, AML	Pulmonary proteinosis, mucocutaneous warts, atypical infections (especially mycobacterial), lymphedema, deafness	Early transplantation for prevention of recurrent infections and pulmonary phenotypes	(45, 46, 47)
*RUNX1*	MDS, AML, B/T-ALL	Thrombocytopenia, platelet dysfunction	Clinical manifestations arise at younger ages in subsequent generations; Consideration of early HCT given poor outcomes for RUNX1 mutant leukemia	(48, 49)
Short Telomere Syndromes	Bone marrow failure, MDS, AML	Reticular skin pigmentation, pulmonary fibrosis, dystrophic nail changes, cirrhosis	Mitigation of pulmonary toxicity by avoidance of busulfan-based conditioning	(50, 51)
*TP53*	MDS, AML, B/T-ALL, lymphomas	Sarcomas, brain tumors, carcinomas of the GI-tract, breast, adrenal cortex, and lung	No precision medicine approaches; High rates of relapse after transplant	(31)

Hematopoietic and non-hematopoietic phenotypes of well-recognized HHMs are described, with preference given to HHMs for which published experiential data exist for allogeneic transplantation. Fanconi genes include: FANCA, FANCB, FANCC, FANCD1 (BRCA2), FANCD2, FANCE, FANCF, FANCG, FANCI, FANCJ (BRIP1), FANCL, FANCM, FANCN (PALB2), FANCO (RAD51C), FANCP (SLX4), FANCQ (ERCC4), FANCR (RAD51), FANCS (BRCA1), FANCT (UBE2T), FANCU (XRCC2), and FANCV (REV7). Short telomere syndrome genes include DKC1, NAF1, TERC, TERT, RTEL1. “? “ indicates a suspected disease phenotype.

## Patient-level implications pre- and post-transplant

As mentioned above, proper HHM evaluation, ideally concurrent with initial workup of hematologic malignancy, provides both diagnostic and clinically actionable information that may prove highly relevant to the patient’s course. Expert protocols and recommendations guiding HHM evaluation have been described previously (52, 53). Although it has been established that germline variants may be inferred from tumor-only somatic variant panel testing (54, 55), a proper HHM workup involves sampling non-tumor tissue. Skin biopsies are preferred, as other sources such as saliva may be contaminated with WBC harboring somatic variants and thereby obscuring variant adjudication (56). Due to increasing awareness of HHMs amongst patients and providers, commercial options offering genetic testing specifically for hereditary blood cancer evaluation have been developed and are currently marketed to patients. However, a high degree of heterogeneity exists within the commercial HHM testing market. In addition to differences in cost, turnaround time and variant validation methods, important genes are missing from commercial many commercial panels and companies accept a wide range of tissues as representative germline samples, including peripheral blood and/or bone marrow specimens (57). Furthermore, most panels are not routinely updated in a timely manner to reflect evolving literature (58).

Donor cell leukemia (DCL) is a catastrophic yet often avoidable consequence of allogeneic transplant whereby pathogenic donor clones expand in the recipient and contribute to apparent “relapses.” This phenomenon may more accurately be described as donor-derived hematopoietic malignancy since the initial neoplasm is not truly relapsing from a dormant state, but rather, a new hematologic cancer is propagating. Prior to advent of commercially available NGS, cytogenetic testing was used to identify donor cell leukemias (DCL) as a complication of allogeneic HSCT (59). Early series estimated the incidence of DCL was initially estimated to be as high as 5% (60). However, it is now thought of as a rare complication of allogeneic stem cell transplantation, with multiple studies suggesting an incidence below 1% (61–63). Despite the limited incidence, overall outcomes of reported DCL cohorts have suggested a poor prognosis with a high mortality rate after diagnosis (61, 64). Cases of donor cell MDS has also been reported after matched-related donor transplant, suggesting the possibility of germline variant carriage in the family member ultimately used as a stem cell source (65, 66). Some reports for which donor follow-up was available have also described the clinical trajectory of hematopoietic malignancy development in the donor after donor-derived relapse in the recipient (67). Outcomes for donor cell MDS, while sparsely reported, also appear to be poor, with published institutional experiences underscoring the importance of proceeding to HCT where possible (68).

Several mechanisms have been suspected to enable leukemic transformation of donor-derived cells (61, 69). Instances of germline mutations present in the hematopoietic cells of apparently healthy donors are well-described (70–72). Due to the potentially elevated risk of developing DCL in recipients of cells with mutated germline predisposition alleles, screening and consideration for exclusion of these donor cells has been suggested (62). Donor screening is of particular importance when using related donors and when there is high probability for HHM, for which ample suspicion and appropriate evaluation is critical before allograft (73). One barrier to comprehensive donor HHM might stem concerns regarding turnaround time of NGS (typically several weeks) and the possible need to delay of HCT. However, if a pathogenic variant has been identified in the transplant recipient, panel-based NGS in a donor candidate is likely unnecessary. We prefer direct single-gene testing for the gene in question from the peripheral blood. Although peripheral blood should not be the source of germline sampling in a patient with hematopoietic malignancy, it is appropriate for evaluation of healthy donors.

There are also ethical challenges arising from donor DNA sequencing after allogeneic HSCT. These considerations are of importance in cases of DCL, where donor germline variants can be detected and potentially carry significant clinical implications for the donor (74, 75). Moreover, variants of uncertain significance can also complicate the interpretation of the results. These scenarios raise ethical questions regarding test result disclosure to the donor. These issues are further exacerbated in cases of unrelated donors, where communication with the donor is limited, and in cases involving cord blood HSCT, where the donor is of minor age (76). If pursued, communication with these donors is likely best facilitated by involvement of agencies that support transplant programs (National Marrow Donor Program, DKMS).

## HHM transplant evidence and outcomes

Patients with HHMs receive similar therapies to those with *de novo* disease often because the underlying causative germline variant is not discovered until after treatment has been initiated (5, 77). STS discussed above, develop due to germline mutations in telomeric regulators that affect telomere structure and function. Patients who develop bone marrow failure or MDS often go on to receive HCT (78). Selection of an appropriate chemotherapy conditioning regimen is important for STS due to a noted propensity for affected patients to develop organ failure as part of their underlying genetic syndrome. In STS, these non-hematologic disease manifestations include pulmonary fibrosis and cirrhosis (50, 79, 80). For example, in patients with dyskeratosis congenita (DC), high rates of death due to pulmonary toxicity have been observed in patients who received busulfan-based conditioning regimens. Fludarabine-containing reduced intensity conditioning regimens are becoming standard of care for these patients, having been shown to be safe and effective without accelerating pulmonary damage (51, 81).

Another example of a precision-medicine guided approach to HCT in patients with HHM pertains to mitigation of post-transplant graft-versus-host disease. Patients with germline *DDX41* germline variant have been shown to have higher rates of both severe (stage 3-4) acute graft-versus-host disease (GVHD) (38%) and moderate to severe chronic GVHD (33%) compared to those without any identified P/LP germline variant (9% and 10%, respectively) (36). GVHD prophylaxis with cyclophosphamide has been shown to prevent development of GVHD in patients with DDX41 germline variants. For example, among 15 patients with P/LP germline *DDX41* variants who underwent HSCT, but did not receive post-HSCT cyclophosphamide, 7 deaths occurred, 4 of which were due to severe GVHD. Five patients with P/LP germline *DDX41* variants who received post-HSCT cyclophosphamide were completely free of GVHD and alive at the time of analysis (36). Though the mechanism for cyclophosphamide-mediated GVHD amelioration has not been delineated, *DDX41* contributes to STING signaling and may lead to aberrant immune activation after transplant (82, 83).

As mentioned above, GATA2 deficiency, stemming from pathogenic variants in GATA2, is comprised of a syndrome characterized by life-threatening opportunistic infections with non-tuberculous mycobacteria (NTM), fungal infections, or human papillomavirus (HPV) infections and a propensity to progress to MDS, CMML, and AML (17–19). Because of immunodeficiency in multiple cellular compartments, the pursuit of HCT in GATA2 deficiency is aimed at addressing both bone marrow hematopoietic stem cells predisposed to developing myeloid malignancy, in addition to restoring functional immune compartments and avoiding deadly consequences of recurrent pulmonary infections. Accordingly, HCT has been shown to be highly effective at preventing recurrent NTM, with zero patients experiencing recurrence of disease following transplant in a 14-patient cohort treated at the National Institutes of Health (45). Risk for post-HCT HPV infections, however, does not appear to be fully addressed by transplantation, with about 50% of patients showing persistent mucocutaneous warts (84). Furthermore, pulmonary complications, including pulmonary hypertension and alveolar proteinosis appear to be substantially mitigated with HCT (85).

Although HCT is an effective means at mitigating, or even reversing clinical phenotypes associated of GATA2 deficiency, the optimal timing of HCT, and choice of conditioning regimen, donor sources, and GVHD prophylaxis remain areas of active investigation. With the potential to mitigate or avoid future syndromic disease manifestations, preemptive HCT in patients with GATA2 deficiency represents an attractive strategy. From 2013 to 2020, 59 patients underwent HCT with busulfan-based conditioning at the National Institutes of Health. Patients were eligible for transplant if they had at least one episode of a life-threatening opportunistic infection, with almost half of patients having received a transplant due to an established diagnosis of MDS or the presence of cytogenetic changes in the absence of overt morphologic disease, particularly monosomy 7. Overall survival (OS) and event-free survival (EFS) at 4 years were 85.1% and 82.1% respectively (86). It is important to note that malignant myeloid progression is possible after HCT, given the malignant clones can propagate in the setting of MDS. Because GATA2-deficient bone marrow stem cells are at a proliferative disadvantage (45), nonmyeloablative conditioning regimens have also been pursued. These regimens have largely consisted of fludarabine, alemtuzumab and busulfan, which have been shown to be an effective strategy. No grade III-IV acute GVHD was observed in patients who received haploidentical related donor HSCTs and prophylaxis with post-transplantation cyclophosphamide followed by tacrolimus/mycophenolate. Ninety-six percent of these patients had complete reversal of the hematologic disease phenotype by one-year post-transplant (86).

**Figure 2:**
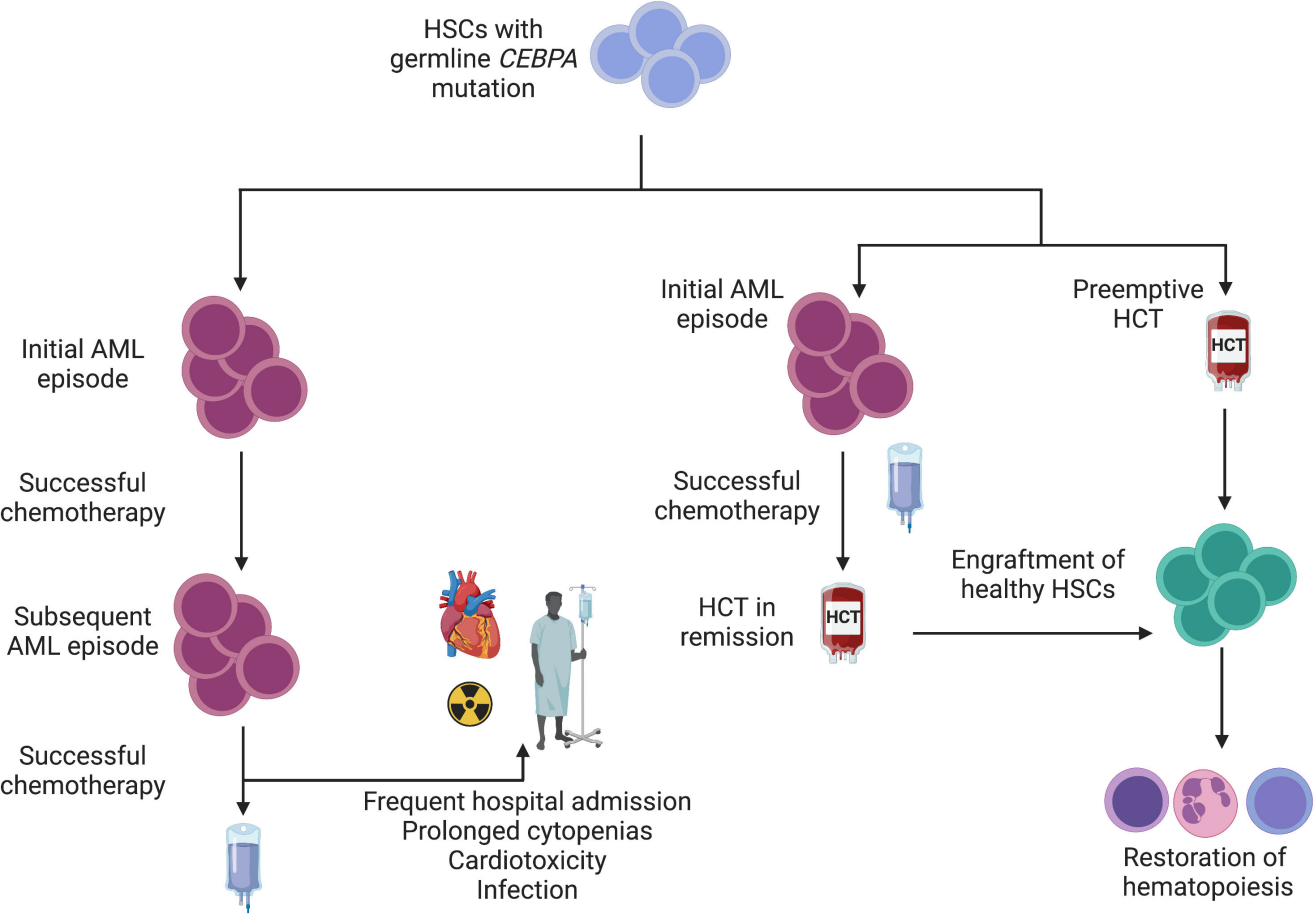
Interventions for familial *CEBPA*-mutated AML. Given a young age at diagnosis and a high likelihood of AML development among variant carriers, a schematic for management is depicted. Individuals who wish to avoid HCT or for whom HCT is not feasible may achieve disease control with chemotherapy alone. Failure to correct a mutated stem cell pool (left) sets the stage for subsequent leukemic episodes. Although long latency periods between subsequent episodes have been observed (15), repeated exposure to cytotoxic chemotherapy confers risk of infection and organ toxicity. In families with highly penetrant disease (right), empiric HCT is reasonable to mitigate AML development and ensure normal hematopoiesis. Alternatively, HCT can be pursued after initial remission is achieved following an initial AML episode.

HCT has also been shown to be an effective strategy for familial *CEBPA*-mutated AML. This syndrome is associated with high penetrance and a young age of AML onset without development of preceding myelodysplasia. Patients typically experience complete response to induction chemotherapy with standard regimens (15). Indeed, the rate of achieving a first CR in a seminal study of 24 individuals spanning 10 families of familial AML was 91% (n = 21/23). Disease recurrence was common, however, with the cumulative incidence of relapse being 56% at 10 years. Although recurrent leukemic episodes remain generally responsive to chemotherapy, treatment-related cardiotoxicity has been observed. Of the 7 patients who died following recurrence of disease, 71% (n = 5) were in remission. One patient had treatment-related complications (cardiotoxicity) and 3 patients had transplant-related complications (GVHD, post-transplant lymphoma, and infection). It is difficult to balance the risk of transplant-related morbidity/mortality with the risk of organic and infectious complications due to cyclical treatment of recurrent leukemic episodes. Preemptive HCT in known variant carriers has been proposed, but data are lacking to widely support empiric HCT vs. proceeding to allograft after appropriate donor evaluation once remission has been achieved (**Figure 2**) (87).

## Conclusions

The study of HHMs has advanced substantially in recent years due to progress in NGS technologies, their clinical integration, and cost accessibility. These technological achievements have enabled physicians and scientists to partner with motivated patients and uncover novel HHM genes, define mechanistic principles that underlie the stepwise progression towards overt malignancy, define the presence or absence of precursor states, and characterize clinical trajectories. Future research in the field will be aimed at optimizing protocols for clinical surveillance in unaffected variant carriers. As clinical integration of NGS in has dramatically increased the number of HHM patients identified, secondary referrals for their family members have also increased the number of clinically unaffected variant carriers seeking consultation. Thoughtful intervention in high-risk HHM variant carriers is also likely to be an area of exciting investigation. Decision-making models to define the optimal time for intervention are needed. Preemptive HCT remains an attractive option for patients with HHM variants associated with deleterious systemic clinical phenotypes (GATA2 deficiency, for example), but non-relapse mortality remains a serious obstacle towards more uniform deployment of potentially curative strategies to replace deleterious stem cell populations. Furthermore, as gene therapy approaches advance, less invasive means for correcting pathogenic variants may become feasible.

## Author contributions

GR conceived the article. TO’C, RS, RM-M, and GR researched and wrote the manuscript. All authors edited the manuscript. GR created figures included in publication. All authors contributed to the article and approved the submitted version.
